# Gender Differences in Social Embeddedness Determinants of Loneliness Among Moroccan and Turkish Older Migrants

**DOI:** 10.1093/geronb/gbad177

**Published:** 2023-12-18

**Authors:** Rowan L F ten Kate, Tineke Fokkema, Theo G van Tilburg

**Affiliations:** Department of Sociology, University of Groningen, Groningen, The Netherlands; Department of Sociology, University of Utrecht, Utrecht, The Netherlands; Netherlands Interdisciplinary Demographic Institute (NIDI)-KNAW/University of Groningen, The Hague, The Netherlands; Department of Public Administration and Sociology, Erasmus School of Social and Behavioural Sciences, Erasmus University Rotterdam, Rotterdam, The Netherlands; Department of Sociology, Vrije Universiteit Amsterdam, Amsterdam, The Netherlands; (Social Sciences Section)

**Keywords:** Gender roles, Loneliness, Migration, Social ties

## Abstract

**Objectives:**

Moroccan and Turkish migrants residing in Northwestern Europe have high loneliness levels. This study examines gender differences in loneliness within this migrant population. The migrants have gender-segregated social roles at home and in public, which might lead to gender differences in what aspects of social relationships can explain variation in loneliness.

**Methods:**

Respondents are from the Longitudinal Aging Study Amsterdam with 446 first-generation Moroccan and Turkish migrants in the Netherlands, aged between 55 and 66 years. We use interaction effects to test for gender differences in determinants of loneliness.

**Results:**

Men and women have a similar, moderate level of loneliness. Having a spouse and receiving care from children are more strongly related with lower loneliness levels in men than in women. Coethnic ties play an equally important role for men and women. In men, frequent mosque attendance is related with greater loneliness, but not in women.

**Discussion:**

Family ties are more protective against loneliness for older men than for older women, possibly indicating that migrant women’s expectations regarding family go above and beyond having a spouse, receiving intergenerational care, or having frequent contact with children. In addition, migrant older men’s higher expectations regarding a public social life could make their social life in the Netherlands less fulfilling, resulting in greater loneliness.

The aging migrant population in the Global North has a relatively high loneliness (Fokkema & Ciobanu, 2021; Wu & Penning, 2015), especially those with a different linguistic and cultural background compared to the majority population (de Jong Gierveld et al., 2015). This applies, for instance, to the large group of Moroccan and Turkish older migrants in Europe (Fokkema & Naderi, 2013; van Tilburg & Fokkema, 2021). Their high level of loneliness can be explained by low socioeconomic status and poor health (van Tilburg & Fokkema, 2021), and low host language proficiency and experienced ethnic discrimination (Cela & Fokkema, 2017). However, little is known about differences in loneliness *within* the diverse Moroccan and Turkish migrant population (Fokkema & Ciobanu, 2021). Loneliness is related to physical and mental health problems (Hawkley & Cacioppo, 2010), and reveals the unpleasantness of deficits in the quantity or quality of one’s social relationships (Perlman & Peplau, 1981).

In this study, we focus on gender roles of Moroccan and Turkish migrants and its consequence for loneliness in order to better understand why migrant men and women feel lonely. More specifically, we focus on having family and nonfamily ties and formal social participation, that is, being a member of organizations or active in paid and volunteer work. Expectations for social embeddedness differ between men and women, consequently affecting loneliness (Dykstra & de Jong Gierveld, 2004). This implies that explanatory factors of loneliness may differ between men and women. For Moroccan and Turkish migrants, gender differences are also expected given the social roles focused on family for women and on public social life for men (Arends-Tóth & van de Vijver, 2008). However, potential gender differences related to loneliness among migrants have not been studied before. We therefore address the research question: What gender differences are there in social embeddedness determinants of loneliness for Moroccan and Turkish older migrants living in the Netherlands?

## Study Context

Moroccan and Turkish older migrants represent two of the largest migrant groups in the Netherlands as they form 20% of the overall first-generation migrant population aged 55 and older (Statistics Netherlands, 2022). In this study, the term first generation refers to persons born in Morocco or Turkey who came to the Netherlands in the years 1960–1980, to find employment (often men) and for reasons of family formation or reunification (often women; Lucassen & Penninx, 1997). Compared to their native-born Dutch-age peers, Moroccan and Turkish migrants have more frequent and more severe health problems, leave the labor market earlier, and experience worse social-economic conditions (Conkova & Lindenberg, 2018). A majority of Moroccan and Turkish older migrant women has never had a paid job (Huschek et al., 2011; Pels, 2000) and often have a lower host language proficiency than migrant men (Schellingerhout, 2004).

## Culture and Gender Roles

The culture of the country of origin of first-generation Moroccan and Turkish migrants differs from the dominant culture in the Netherlands in two ways. First, Moroccan and Turkish migrants come from collectivistic countries (de Valk & Schans, 2008). Collectivistic cultures value family obligations highly and put the interests of family and community before self-interest (Baykara-Krumme & Fokkema, 2019). Individualistic cultures, such as contemporary Dutch culture, involve fewer obligations toward family relationships and place self-interest above that of family and community (Pels & de Haan, 2007; Taniguchi & Kaufman, 2022). Second, the majority of first-generation Moroccan and Turkish migrants originate from rural areas with patriarchal value orientations, implying that family obligations differ between men and women. Obligations for women translate into responsibility for the household and childcare. For instance, migrants living in the Netherlands originating from patriarchal cultures have gendered care roles where women are expected to provide (hands-on) family care (Ahmad, 2022). Obligations for men are to be the decision makers of the household (Pels, 2000), including household finances, and matters outside the household (Ataca et al., 2005). Thus, women and men have different and segregated roles both at home and in public (Huschek et al., 2011).

First-generation Moroccan and Turkish migrants tend to maintain their collectivistic and patriarchal values (Arends-Tóth & van de Vijver, 2008; de Valk & Schans, 2008). To some extent, the collectivistic and patriarchal values are interrelated because they both emphasize the importance of obligations toward the family. However, whereas both in individualistic and collectivistic cultures, women tend to take primary responsibility for caregiving (Swinkels et al., 2019), this gender role is even more pronounced in patriarchal cultures. In Dutch society, there is less of a gender-based hierarchy in decision making and in the division of (care) roles at home and in public (Arends-Tóth & van de Vijver, 2008). The collectivistic and patriarchal values also coincide with the Islamic religion in the countries of origin such as children’s duties to be respectful to their parents (Nhass & Verloove, 2020). However, because these values are not bound to just the Islamic religion, we focus on collectivistic and patriarchal values as broader indicators of culture.

While collectivistic values focus mainly on family obligations, patriarchal values affect gender norms in social ties in and outside the family and in formal social participation. Based on the distinction in gender roles according to patriarchal culture, we categorize our hypotheses accordingly. For Moroccan and Turkish migrant women, the overall expectation is that they are involved in the nuclear family life, especially with regards to intergenerational ties. For Moroccan and Turkish migrant men, the overall expectation is that they are involved in public social life and contribute to the household through income from paid employment and decision-making roles. The gender roles contrast with findings from research among nonmigrants in the Netherlands, where a diverse and large social network is more important for women than for men (Dykstra & de Jong Gierveld, 2004). Given the similarities of the above cultural aspects of country of origin for Moroccan and Turkish migrants, we do not distinguish by country. For the sake of readability, we use migrants to refer to both Moroccan and Turkish older migrants.

## Hypotheses for Family Ties

### Spouse

Marriage in collectivistic cultures is generally centered around economic roles and gaining social status, as opposed to marriage as an emotionally fulfilling social institution that protects against loneliness in individualistic cultures (Taniguchi & Kaufman, 2022). Among people from individualistic cultures, men were found to be more dependent on their spouse than women in terms of the fulfillment of their social needs (Dykstra & de Jong Gierveld, 2004). For migrants, we assume the same differential gender effect based on patriarchal values. This is because women can find fulfillment in the role of family caretaker (Palmberger, 2019; Pels, 2000), regardless of whether they have a spouse. For men, who place a high importance on being the financial caretaker for the household and having the authoritative role as husband (Pels & de Haan, 2007), having a spouse has more importance. Hypothesis 1 is that having a spouse is more protective against loneliness among migrant men than among migrant women.

### Children

Migrant women, as a result of patriarchal values, place high value on the role of parenthood (Pels, 2000). As a consequence, a frequent contact with children is not only highly valued by women but should potentially indicate more often a high-quality relationship among women as compared to women with infrequent intergenerational contact. Migrant father–child relationships are more defined by patriarchal values (Ataca et al., 2005). A high level of intimacy with children may even be seen as damaging to fathers’ patriarchal role (Pels, 2000). Hypothesis 2 is that more frequent contact with children is more protective against loneliness among migrant women than among migrant men.

Next to contact frequency, intergenerational ties can involve care. In collectivistic cultures, parents have high filial expectations that their children will provide care to them in later life (de Valk & Schans, 2008; ten Kate et al., 2021). This expectation has a gendered dimension within patriarchal culture where women, as opposed to men, are expected to provide intergenerational care (Ahmad, 2022). For migrants, care is preferably given by children and not by healthcare professionals (Conkova & Lindenberg, 2020; Palmberger, 2019). However, children who grew up in the residence country may not adhere to the expectations of their parents, resulting in parental disappointment (Kalmijn, 2019; Tezcan, 2018). Some research suggests that fathers especially endorse high filial expectations (Conkova & Lindenberg, 2018, 2020). Other research suggests that mothers have high expectations because they take for granted that children will reciprocate the care (Kagitcibasi & Ataca, 2015). In this sense, care is an extension of the close mother–child relationship that is expected for women according to inherited patriarchal gender roles. Moreover, given the reciprocal nature of intergenerational care, women—more than men—might also value receiving intergenerational care because of their understanding of the burdensome role it can entail (Krobisch et al., 2021). Hypothesis 3a is that receiving intergenerational care is more protective against loneliness among migrant women than among migrant men and Hypothesis 3b is that wanting to receive intergenerational care is more detrimental to loneliness among migrant women than among migrant men.

## Hypotheses for Nonfamily Ties and Formal Social Participation

### Coethnic Ties

Coethnic nonfamily ties are important sources of emotional support, belonging, and companionship among older migrants (Cela & Fokkema, 2017), for both male and female migrants (Bjerke, 2020). Having contact with coethnic peers is associated with less loneliness (Cela & Fokkema, 2017; Salma et al., 2018). A frequent contact with coethnic peers, which are self-selected social ties, should indicate supportive ties among both men and women. Whereas men often meet their contacts outside the home and women meet people at home (Bilecen, 2019; Nhass & Verloove, 2020), the functioning of the contact with coethnic peers should be similar for both men and women. Based on this theoretical reasoning, no gender difference is to be expected in the importance of contact with coethnics. Accordingly, we refrain from formulating a hypothesis, but leave the possibility open for a differential gender effect in the protective effect of more frequent contact with coethnic, nonfamily peers against loneliness.

### Formal Social Participation

Social participation in activities of formal organizations, such as mosques and neighborhood centers, provides support and a sense of belonging, and is important for migrants’ well-being (Palmberger, 2019). Moreover, through paid work people get a large and diverse social network, which reduces loneliness (ten Kate et al., 2020). However, when focusing on just the setting of the contact rather than the emotionally supportive social ties in such a setting, we expect that formal social participation creates social status outside the household, which is especially valued by men (Böcker & Gehring, 2015; Conkova & Lindenberg, 2020). Women may value social embeddedness in a private setting more than formal social participation in a public setting. Migrant men, following patriarchal roles, are expected to engage in activities outside the household such as paid work. In turn, men place higher value on their embeddedness in public social life in order to fulfill expected gender roles. Hypothesis 4 is that formal social participation is more protective against loneliness among migrant men than among migrant women.

## Covariates

### Transnational Ties

Transnational ties to the country of origin can have an ambivalent impact on one’s well-being (Klok et al., 2017). On the one hand, maintaining a connection with the country of origin accommodates negative experiences in the country of immigration (Horn & Fokkema, 2020). On the other hand, transnational ties increase loneliness as the overall sense of belonging is reduced to one place—what is known as “uprootedness” (Klok et al., 2017). In the latter scenario, transnational ties reinforce missing out on social life in the country of origin and evaluating life negatively in the country of immigration, resulting in more loneliness (Horn & Fokkema, 2020).

### Resources

We control for various resources that can protect against loneliness among older migrants, namely a good health, socioeconomic status, language proficiency, and sense of mastery over life (Cela & Fokkema, 2017; van Tilburg & Fokkema, 2021). These resources can help to expand the social network beyond family ties and also make it easier to maintain existing social ties.

Furthermore, we include age. As a control for intergenerational ties, we include the number of persons in the household besides the spouse and the number of children.

## Method

### Sample

Data were from the Longitudinal Aging Study Amsterdam (LASA) with *n* = 446 migrants (259 men and 187 women) born in Morocco or Turkey who live independently and have children (Hoogendijk et al., 2020). Data were collected in 2013 and 2014; 191 persons of Moroccan origin and 255 of Turkish origin were interviewed. Respondents were aged between 55 and 66 years. On average, they migrated 35.7 years before (*SD* = 7.4). Given that Moroccan and Turkish migrants tend to leave the labor market earlier and experience health problems at a younger age, the term old is more applicable to migrants of this age group as compared to native Dutch.

The samples were stratified by gender and taken from municipal population registers of cities with population sizes between 85,000 and 805,000. The data were collected at respondents’ homes by trained interviewers using standardized questionnaires. The respondent’s migration background was also matched with the interviewer’s background. The cooperation rate, defined as the number of completed interviews divided by the total number of contacted eligible persons, was 0.45. The languages of the interviews were Dutch, Moroccan-Arabic (Darija), Berber (Tarifit), or Turkish. For various questions the translations were taken from previous research, such as the loneliness scale (Uysal-Bozkir et al., 2017). Questions unavailable in Moroccan-Arabic, Berber, or Turkish were translated by two professionals using the back-translation method.

### Instruments

#### Loneliness

Loneliness (Kuder- Richardson (KR) = R-20 = 0.83) was measured by the 11-item De Jong Gierveld scale, a valid tool for measuring loneliness among older migrants (Uysal-Bozkir et al., 2017; van Tilburg & Fokkema, 2021). The scale has six negatively formulated items such as “I experience a sense of emptiness” and five positively formulated items such as “There are enough people I feel close to.” Responses were summed with “yes” and “more or less” counting as lonely on the negatively formulated items, and “more or less” and “no” counting as lonely on the positively formulated items.

#### Family relationships

Respondents were asked if they had a spouse (1 = yes) and others (1 = yes) living in the same household. Unfortunately, it is not known for all respondents whether coresidents were respondent’s children because this question was included in another interview in which not all respondents participated. For the 356 respondents with data, only four did have others in their homes who were not children. Thus, although we do not know whether all coresidents of respondents are (adult) children, it seems likely that the variable indicates some degree of (intergenerational) support in the household. With these two variables, the reference group in the multivariate regression model is respondents who live alone. The total number of children that respondents have was also included. Contact frequency was asked for nonresiding children. Response options ranged from 1 (never or less than once a year) to 5 (daily).

Respondents indicated whether someone helped with their personal care, personal care of the spouse, domestic tasks, or nursing tasks. Given our focus on children, we included whether care was received from children (1 = yes). Respondents also indicated whether care was sufficient. If that was not the case, respondents could indicate that they would like to receive care from a child (1 = yes; this could be in addition to care already provided by a child).

#### Nonfamily ties and formal social participation

Contact frequency was included for Moroccan or Turkish friends and acquaintances and Moroccan or Turkish neighbors. For each type, the response options ranged from 1 (never or less than once a year) to 5 (daily). Respondents who answered “not applicable” were given the value of 1 given that our focus is on the frequency of contact rather than the existence of coethnic ties. For coethnic ties, we used the maximum frequency score on the variables for friends and acquaintances, and neighbors, given that these contacts can be mutually interchangeable. For formal social participation, three indicators are used. First, we included whether respondents are active members or involved in an organization, with the exclusion of religious organizations or mosques. Second, frequency of visits to the mosque was measured as (1) once a week or more and (0) a few times a month or less often. Religious organizations were excluded from the first indicator because of the strong association with mosque attendance frequency (*r* = 0.61). Third, participation in the labor market (i.e., paid work at the time of the interview) was included (1 = yes).

#### Covariates

Two indicators measure an evaluation of transnational ties and one identifies a behavioral component. First, feelings of loss concerning the country of origin were measured by five items from the Lowlands Acculturation Scale (Mooren et al., 2001): “I belong here less than in Morocco/Turkey”; “Although I live here, it does not feel as my country”; “Morocco/Turkey is always in my mind and in my memories”; “I miss the people I left behind in Morocco/Turkey”; “I am homesick.” Responses were 0 “no” and 1 “yes,” and were summed (KR-20 = 0.73). Second, respondents indicated whether they considered return migration (1 = yes). Third, a dichotomous variable indicated whether respondents had visited the country of origin in the past year for a continuous duration of at least 2 months (1 = yes).

Four resources are included. First, Dutch language proficiency was measured by a rating from 1 (strongly disagree) to 4 (strongly agree) on the agreement with three statements about the ability to understand or speak Dutch. Values were summed for the scale (α = 0.84). Second, two measures of socioeconomic status resources were included: educational level ranged from 1 (primary school not completed) to 9 (university level); satisfaction with income ranged from 1 (dissatisfied) to 5 (satisfied). Third, perceived health ranging from 1 (poor) to 5 (excellent) was included. Fourth, mastery (Pearlin & Schooler, 1978) was included, which is a sum score over five items (α = 0.86) with responses ranging from 1 (strongly agree) to 5 (strongly disagree). An example item is: “I have little control over things that happen to me.”

#### Hypotheses testing

A generalized linear regression model was estimated with interaction dummies. We present our main analyses to include only respondents who have children in order to test Hypotheses 2, 3a, and 3b that assume respondents have children. However, given that Hypotheses 1 and 4 do not rule out the possibility that respondents are childless, we conducted a sensitivity analysis including respondents who do not have children (*n =* 13). The findings did not deviate (Supplementary Table S1).

#### Missing values

The percentage of missing values was below 5% on all variables. Missing values on the multivariate regression model were more likely for women than for men (65% vs 35%). Multiple imputation with 20 imputations was used to account for possible bias of missing values.

## Results

### Description of Gender Differences

Table 1 displays an overview of gender differences in variable distributions. On average, men and women have a similar, moderate level of loneliness (average score is between 3 and 8). Of the sample, 24% is not lonely (score 0–2) and 20% has a severe level of loneliness (score ≥ 9). Concerning family ties, compared to men, women coreside less often with a spouse and with others but have a more frequent contact with children ties than men. As for intergenerational care, more women receive care from their children than men, but more women also want to receive (more) care from their children.

**Table 1.** Gender Differences in Variable Distributions^a^

Variable	Men		Women		*t*/Chi^2^
	*N* ≤ 259		*N* ≤ 187		
	*M*	*SD*	*M*	*SD*	
Loneliness (0–11)	5.2	3.3	5.2	3.2	−0.2
Has a spouse in the household (0–1)	0.88		0.68		25.6***
Presence of others in the household (0–1)^b^	0.66		0.58		3.4
Number of children (1–13)	4.0	1.9	4.3	2.0	−1.3
Frequency of contact with nonresident children (1–5)	4.2	0.8	4.5	0.7	−4.6***
Received care from children (0–1)	0.12		0.28		18.9***
Wants (more) care from children (0–1)	0.04		0.10		5.1*
Contact frequency with coethnic ties (1–5)	4.3	0.9	3.9	1.0	4.1***
Participation in organization (0–1)	0.29		0.34		1.6
Mosque attendance once a week or more (0–1)	0.72		0.34		65.5***
Paid work (0–1)	0.33		0.10		33.8***
Feelings of loss (0–5)	3.4	1.5	3.5	1.5	−0.5
Return migration considerations (0–1)	0.27		0.24		0.6
Long-stay visits to Morocco/Turkey (0–1)	0.18		0.30		7.9**
Turkish origin (vs Moroccan)	0.55		0.60		1.4
Age (55–66)	60.9	3.0	60.9	3.0	−0.1
Dutch language proficiency (3–12)	7.7	2.3	6.8	2.3	4.1***
Educational level (1–9)	2.9	2.1	2.0	1.5	5.4***
Income satisfaction (1–5)	2.2	1.4	2.5	1.5	−2.1*
Perceived health (1–5)	2.7	1.1	2.3	0.9	3.8***
Mastery (5–25)	15.6	5.2	14.1	5.3	2.9**

*Notes*: *SD* = standard deviation.

^a^Data source: Longitudinal Aging Study Amsterdam (LASA). *n =* 446.

^b^Of the subsample of respondents (*n* = 342) who responded who their household members are, all other coresidents are their children.

*p < .05. **p < .01. ***p < .001.

Men have on average more frequent contact with coethnic ties, although the majority of both men and women have at least weekly contact with coethnic ties. Regarding formal social participation, no gender differences were found in participation in nonreligious organizations. The frequency of mosque attendance is higher for men than for women. More men than women do paid work.

### Hypotheses Testing

**Figure 1.**
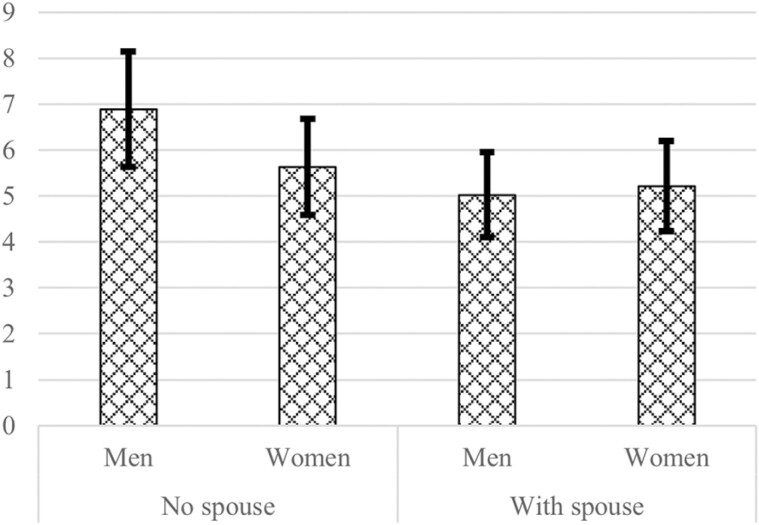
Marginal predicted means of loneliness (0–11) with 95% confidence interval conditioned on marital status, setting all other predictors and covariates at their means, derived from the regression model. Data source: LASA. *n =* 446.

Table 2 presents the pooled multivariate regression model of loneliness including hypothesized gender interactions. Hypothesis 1 found support: among men, the effect of having a spouse is more protective against loneliness than among women. This effect (illustrated in Figure 1) shows that men have a higher estimated level of loneliness than women when they do not have a spouse. There is no difference in loneliness when they are married. The presence of others (most likely children) at the household and having a higher number of children are not protective against loneliness. No support was found for Hypothesis 2 on the stronger protective effect among women as a more frequent contact with children is related to equally lower loneliness level for men and women.

**Table 2.** Regression of Loneliness (0–11)^a^

Predictor	*B*	*SE B*
Intercept	15.15***	2.87
Gender (1 = female)	−1.91	1.79
H1: Has a spouse in the household (0–1)	−1.78***	0.53
× Gender	1.37*	0.67
Has others in the household (0–1)^b^	0.17	0.28
Number of children (1–13)	−0.08	0.08
H2: Frequency of contact with nonresident children (1–5)	−0.58**	0.22
× Gender	0.02	0.38
H3a: Received care from children (0–1)	−1.46**	0.52
× Gender	1.63*	0.67
H3b: Wants (more) care from children (0–1)	1.69*	0.80
× Gender	−0.57	1.02
Contact frequency with coethnic ties (1–5)	−0.71***	0.18
× Gender	0.14	0.29
H4: Participation in organization (0–1)	−0.22	0.38
× Gender	0.40	0.55
H4: Mosque attendance once a week or more (0–1)	0.90*	0.40
× Gender	−1.28*	0.74
H4: Paid work (0–1)	−0.05	0.37
× Gender	−0.28	0.56
Feelings of loss (0–5)	0.14	0.09
Return migration considerations (0–1)	0.35	0.29
Long-stay visits to Morocco/Turkey (0–1)	−0.27	0.32
Turkish origin (vs Moroccan)	0.50	0.32
Age (55–66)	0.01	0.04
Dutch language proficiency (3–12)	0.01	0.06
Educational level (1–9)	−0.14	0.08
Income satisfaction (1–5)	−0.29**	0.09
Perceived health (1–5)	−0.34*	0.13
Mastery (5–25)	−0.19***	0.03

*Notes*: *SE* = standard error.

^a^Data source: LASA. *n =* 446. Pooled generalized linear regression analysis with eight interaction effects. For the hypotheses, the main effects show the predicted effect for men, whereas the starred interaction effects show the additional predicted effects for women.

^b^Of the subsample of respondents (*n* = 356) who were known to have no or resident children, only four did have others in their homes who were not children.

*p < .05. **p < .01. ***p < .001.

**Figure 2.**
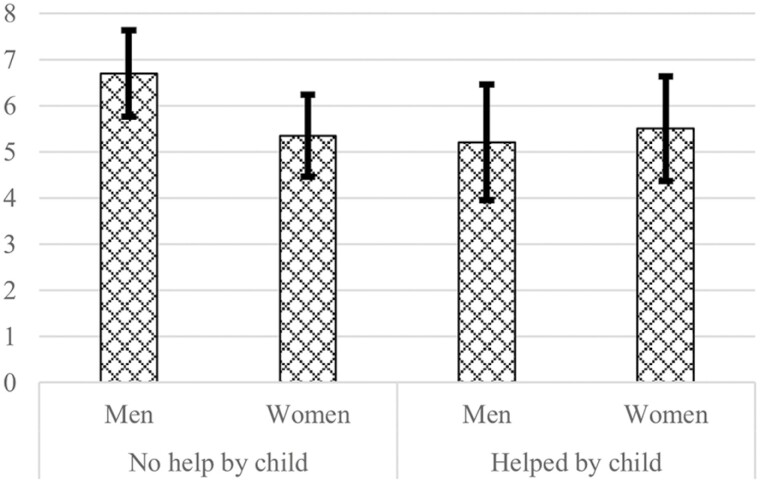
Marginal predicted means of loneliness (0–11) with 95% confidence interval conditioned on receiving help from children, setting all other predictors and covariates at their means, derived from the regression model. Data source: LASA. *n =* 446.

Hypothesis 3a and Hypothesis 3b were not supported either. The opposite direction was found for Hypothesis 3a: receiving intergenerational care is more protective against loneliness among men than among women. Figure 2 displays that women have similar estimated levels of loneliness when they receive intergenerational care compared to when they receive no care. For men, the estimated level of loneliness is lower when they receive intergenerational care than when they receive no care. The desire to receive (more) care from children is significantly associated with more loneliness, but this association did not differ between men and women.

**Figure 3.**
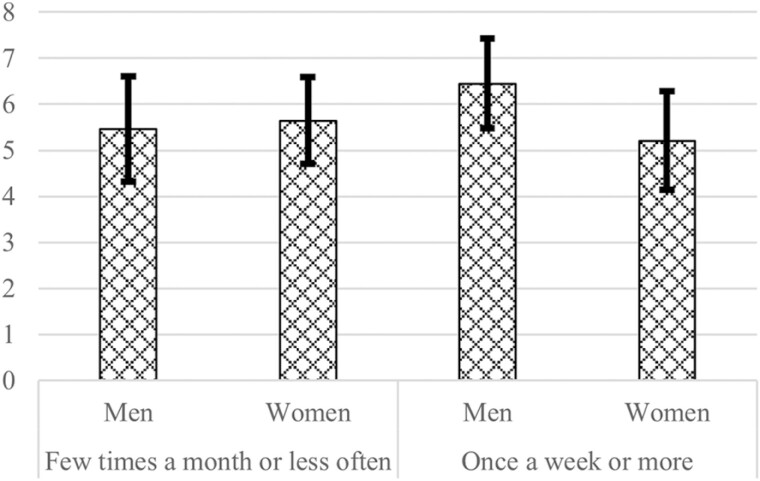
Marginal predicted means of loneliness (0–11) with 95% confidence interval conditioned on mosque attendance frequency (lowest and highest are shown), setting all other predictors and covariates at their means, derived from the regression model. Data source: LASA. *n =* 446.

As expected, there is no gender difference in contact frequency with coethnic ties as a protective resource against loneliness. The findings for formal social participation contrast with Hypothesis 4. Being active in a nonreligious organization and paid work are not associated with loneliness. For mosque attendance, the interaction with gender was in an unexpected direction: frequent mosque attendance is more strongly related to greater loneliness in men than in women. As shown in Figure 3, the estimated level of loneliness with frequent mosque attendance is lower among women than among men.

Indicators of transnational ties were not associated with loneliness. Higher-income satisfaction, perceived health, and level of mastery are protective resources against loneliness. Controlled for all other predictors, country of origin and gender do not predict loneliness.

## Discussion

### Study Findings

Moroccan and Turkish older migrants living in Northwestern Europe have higher loneliness levels than native European populations, yet little is known about the role of gender in explaining loneliness. This specific cohort migrated to Europe for labor and family reunification purposes and generally maintained the cultural values of their country of origin (Arends-Tóth & van de Vijver, 2008; de Valk & Schans, 2008). According to patriarchal gender roles, women are typically expected to look after their family ties, whereas men are expected to look for public social life. We contribute to the literature by showing that some social relationship determinants of loneliness have differential gender effects for Moroccan and Turkish migrants, albeit in some unexpected directions. Hence, in describing gender differences in loneliness within older migrant populations, it is important to consider that the underlying explanations of loneliness can differ between men and women. We expect that the findings on our research population can be applied to migrants of other migration histories who have similar patriarchal values living in Northwestern Europe. This is because migrants generally maintain their cultural values of the country of origin (Baykara-Krumme & Fokkema, 2019), and as a result also maintain gender role expectations.

In support of our first hypothesis, having a spouse is less protective among migrant women than among migrant men, suggesting that women find more fulfillment in other types of social ties. This seems to be a consistent differential gender effect for respondents across individualistic and collectivistic cultures (Dykstra & de Jong Gierveld, 2004; Taniguchi & Kaufman, 2022). In addition, we found that contact with coethnic nonkin has an equally strong protective effect among migrant men and women. This suggests that both migrant men and women value having frequent contact with coethnic peers in order to avoid loneliness. Thus, in cultures where marriage is not necessarily focused on fulfilling emotional needs (Taniguchi & Kaufman, 2022), having supportive ties with one’s peers is of utmost importance. Previous research also indicates that older migrants prefer coethnic ties in receiving emotional support or for discussing certain matters, in part to avoid being a burden to children (Cela & Fokkema, 2017). The experience of minority stress and focus on one’s own culture among migrants (Baykara-Krumme & Fokkema, 2019) can make contact with coethnic peers crucial for preventing loneliness among both men and women.

We expected that the higher value placed on the role of the woman as family caretaker would make intergenerational ties more protective against loneliness (Hypotheses 2 and 3). However, our findings indicate that next to the relationship with the spouse, women’s fulfillment of social needs seems to extend beyond the nuclear family. Given the cultural values placed on women’s role in the family (Ataca et al., 2005; Pels, 2000), migrant women’s expectations might be going above and beyond having frequent contact or receiving care from children. Hence, while receiving intergenerational care and having a higher contact frequency with children can indicate a high-quality relationship among migrant men, this may not necessarily be a satisfying relationship for migrant women. The high expectations toward care also align with our finding that women, more than men, wished to receive (more) care from children. An alternative explanation may be that intergenerational care provided by children might also be undesirable for women who wish to avoid being a burden to their children (Palmberger, 2019). Thus, while receiving intergenerational care is the cultural norm, especially among men (Conkova & Lindenberg, 2020), older migrant women may have shifted their preferences toward receiving formal care in the country of immigration.

Our findings additionally suggest that several types of formal social participation are not protective against loneliness among older migrants, such as involvement in organizations. As indicated by the finding on mosque attendance, this seems to be especially the case for migrant men who have higher expectations regarding public social life than migrant women. The frequent mosque attendance among men may be the result of a lack of alternative social meeting places in the Netherlands (Conkova & Lindenberg, 2020; Nhass & Verloove, 2020), suggesting that the mosque is perhaps the only meeting place for (lonely) migrant men. In addition, the contact in the mosque may be superficial in its content, for instance, by focusing more on religious topics (Nhass & Verloove, 2020) rather than emotional support that reduces loneliness.

### Study Limitations and Directions for Interventions

A first limitation is the small sample size, especially given the number of predictors being examined. A small sample size markedly reduces the statistical power to detect meaningful differences between groups, in our case between men and women.

A second limitation is that the data did not include measures on the quality of the relationship such as emotional closeness with family ties. For instance, some indicators of social ties are measured via contact frequency, which generally indicates a higher quality of the relationship (Bengtson & Roberts, 1991). However, as mentioned before, expectations toward social relationships could concern not only contact frequency but also other indicators such as the quality of the relationship. No other indicators of quality of relationships (e.g., satisfaction, support exchanges, conflicts, satisfaction with social ties during social participation) were available in the present study.

A third limitation is that indicators on social ties to native Dutch were not available. For example, contact frequency was not specified for native Dutch and it was unclear whether respondents had social ties to Dutch members of organizations they partake in. Hence, measures of social embeddedness outside the family were limited to coethnic ties and formal social participation. With regards to men’s desire for social status in public social life in the country of immigration, information on native Dutch would have given insight whether this applies to both coethnic ties and the majority population.

Our findings imply that interventions against loneliness need to address different types of social ties among migrant men and women. For both men and women, coethnic ties are important in reducing loneliness while formal social participation in the country of immigration can be unsatisfactory, especially among migrant men. Nuclear family ties can lower loneliness, but to a lesser extent among migrant women. Therefore, managing migrants’ expectations toward their family relationships and to provide social ties beyond the family, especially among women, seems to be fruitful in reducing migrants’ loneliness. To make formal social participation more meaningful, activities should be offered that provide social support from coethnic friends and fit with migrants’ cultural needs (Salway et al., 2020). In particular, the mosque seems to be of high importance among migrant men, but not a form of formal social participation that is necessarily protective against loneliness. Thus, existing activities at mosques could be extended to provide social interactions among older migrant men that go beyond religious discussions.

## Supplementary Material

gbad177_suppl_Supplementary_Tables_S1
