# Supplementary material for: Gender Differences in Social Embeddedness Determinants of Loneliness Among Moroccan and Turkish Older Migrants
Source: J Gerontol B Psychol Sci Soc Sci. 2023 Dec 18;79(3):gbad177. doi: 10.1093/geronb/gbad177 (PMC10873824; doi:10.1093/geronb/gbad177)
Supplement: gbad177_suppl_Supplementary_Tables_S1 [file gbad177_suppl_supplementary_tables_s1.docx]

**Gender Differences in Social Embeddedness** **Determinants of Loneliness among Moroccan and Turkish Older Migrants**

Supplementary materials

Table S1: Regression of loneliness (0-11) including childless respondents ^a^

|  | B |  | SE B |  |
| --- | --- | --- | --- | --- |
| Intercept | 15.74 | *** | 2.82 |  |
| Gender (1=female) | -1.88 |  | 1.76 |  |
| H1: Has a spouse in the household (0-1) | -1.83 | *** | 0.52 |  |
| * Gender | 1.33 | * | 0.66 |  |
| Has others in the household (0-1) | 0.23 |  | 0.27 |  |
| Number of children (1-13) | -0.08 |  | 0.07 |  |
| H2: Frequency of contact with non-resident children (1-5) | -0.56 | * | 0.22 |  |
| * Gender | 0.03 |  | 0.37 |  |
| H3a: Received care from children (0-1) | -1.44 | ** | 0.52 |  |
| * Gender | 1.60 | * | 0.67 |  |
| H3b: Wants (more) care from children (0-1) | 1.63 | * | 0.79 |  |
| * Gender | -0.50 |  | 1.02 |  |
| Contact frequency with co-ethnic ties (1-5) | -0.66 | *** | 0.18 |  |
| * Gender | 0.14 |  | 0.28 |  |
| H4: Participation in organization (0-1) | -0.30 |  | 0.37 |  |
| * Gender | 0.45 |  | 0.54 |  |
| H4: Mosque attendance once a week or more (0-1) | 0.95 | * | 0.39 |  |
| * Gender | -1.37 | * | 0.56 |  |
| H4: Paid work (0-1) | 0.00 |  | 0.36 |  |
| * Gender | -0.66 |  | 0.70 |  |
| Feelings of loss (0-5) | 0.15 |  | 0.09 |  |
| Return migration considerations (0-1) | 0.34 |  | 0.29 |  |
| Long-stay visits to Morocco/Turkey (0-1) | -0.33 |  | 0.31 |  |
| Turkish origin (versus Moroccan) | 0.51 |  | 0.30 |  |
| Age (55-66) | 0.00 |  | 0.04 |  |
| Dutch language proficiency (3-12) | -0.01 |  | 0.06 |  |
| Educational level (1-9) | -0.12 |  | 0.07 |  |
| Income satisfaction (1-5) | -0.30 | *** | 0.09 |  |
| Perceived health (1-5) | -0.38 | ** | 0.13 |  |
| Mastery (5-25) | -0.19 | *** | 0.03 |  |

* *p* < 0.05; ** *p* < 0.01; *** *p* < 0.001

^a^ Data source: LASA. *n =* 460. Pooled generalized linear regression analysis with eight interaction effects. The main effects show the predicted effect for men whereas the starred interaction effects show the additional predicted effects for women.
